# Allostatic load and canine companionship: a comparative study using biomarkers in older adults^*^

**DOI:** 10.1590/1518-8345.2755.3071

**Published:** 2018-11-14

**Authors:** Alejandro Morales-Jinez, Francisco J. López-Rincón, Alicia Ugarte-Esquivel, Irma Andrade-Valles, Luz Elena Rodríguez-Mejía, José Luis Hernández-Torres

**Affiliations:** 1 Universidad Autónoma de Coahuila, Escuela de Licenciatura en Enfermería, Torreón, Coahuila, Mexico.

**Keywords:** Old Adult, Canine Accompaniment, Allostatic Load, Biomarkers, Cortisol, Stressors

## Abstract

**Objective:**

to compare the biomarkers and the allostatic load levels in a sample of older persons with and without canine companionship.

**Method:**

descriptive and comparative study. Data were collected using a sociodemographic questionnaire and a fasting blood sample. The allostatic load comprised 11 biomarkers that are primary and secondary stress mediators, which arise from the following systems: neuroendocrine, immune, metabolic, cardiovascular and anthropometric.

**Results:**

a significant difference was found in two biomarkers: cortisol (*t=* -3.091, *df*=104, *p=*0.003) and total cholesterol (*t=* -2.566, *df*=104, *p=*0.012), in the allostatic load levels between older adults with and without a canine companionship (*U=* 1714.00, Z= 2.01, *p=*0.044). By associating the allostatic load level with the canine companionship, there was a higher frequency of older adults with low allostatic load among those who have canine companion, compared with those who do not have canine companionship. (χ^2^= 3.69, *df*=1, *p=* 0.043).

**Conclusion:**

canine companionship influences health in a positive way, as the allostatic load is lower in older adults who have a dog as companion, in addition to presenting lower levels of cortisol and total cholesterol.

## Introduction

Human aging, as part of the life cycle, is a complex and multifactorial phenomenon that involves the interrelationship between molecular, evolutionary, socioeconomic, psychological, cultural and social aspects^1^. Although aging affects the functional status and health, there is an effort at international level to improve the quality of life in order to ensure a healthy aging^2^.

According to the World Health Organization, healthy aging is an integral, comprehensive and dynamic process that attempts to maintain the functional capacity as long as possible so that an older person is able to be and do what he considers valuable at this stage of his life. In this sense, functional capacity is not limited only to the physical aspect, it includes the social determinants of health and well-being, life satisfaction, subjective well-being, personal fulfillment, application of appropriate policies and human rights^2^.

It is evident that the social environment plays an important role in healthy aging. The economic context, social networks, unfavorable circumstances in the neighborhood, and even discrimination, interact immediately with the older person and can generate stress, affecting the physical, mental, spiritual and social aspects of older adults, which can trigger chronic diseases^3^. The main stressful circumstances in older adults are the loss of friends and family, economic problems, decreased physical and mental functional capacity, retirement, health problems, feelings of loneliness and isolation, among others^4^ ^-^ ^5^.

When an older person consciously detects a situation as stressful, a biochemical reaction is triggered to achieve allostasis or adaptation to the threat, however, if this adaptation process is not reached, an overload arises. Allostatic overload or allostatic load is defined as accumulated physiological wear and tear that results from poor adaptation to environmental stressors. It involves the abnormal functioning of primary mediators, such as cortisol, and secondary mediators, such as C-Reactive Protein (CRP), fibrinogen, blood pressure, total cholesterol, high density lipoproteins, glycosylated hemoglobin, among others, what is currently considered an evolutionary concept of the term stress^6^ ^-^ ^7^.

On the other hand, the human being as an entity of a social nature, has always sought to relate with other humans or even to different living species, such as animals. One of the preferred species of humans is the canine species, as the fossil remains have shown that dog has accompanied man since ancient times^8^. Animal companionship involves a mutual and meaningful connection with complex physiological and psychological interactions between the person and the animal. Unlike the animal assisted therapy, in which there is a purpose and a trained animal and with specific characteristics, animal companionship occurs at home, in a context of intimacy and proximity to the caretaker, which includes a special treatment involving affection, care and attention^8^ ^-^ ^12^.

Animal companionship and its impact on health have been the subject of scientific studies for several decades; however, there are inconsistencies on this subject. On the one hand, some results indicate that animal companionship has a positive effect on health in several age groups, including older persons, and contrarily, other studies attribute the positive effects on health to other causes that are not associated with animal companionship^12^ ^-^ ^14^.

Among the positive results of the canine companionship on health in older adults are the improvement in health perception and quality of life^12^ ^,^ ^15^ ^-^ ^16^, motivation for the older adult to walk and increase his walking time^17^ ^-^ ^19^ and take a better care of himself when he has a chronic-degenerative disease^20^, there is a decrease in medical visits^18^ ^,^ ^21^, decrease in the feeling of loneliness^19^ ^,^ ^22^ ^).^, in addition to facilitate social interaction, improve empathy and the perception of emotions^21^, help to cope with the loss of a loved one^18^, decrease the perception of stress^11^ and the systolic blood pressure^11^ ^,^ ^21^, there are lower levels of depression and anxiety^19^ and, at a biochemical level, people with a canine companionship have lower levels of cortisol^19^ ^,^ ^21^ ^,^ ^23^, triglycerides and cholesterol^21^.

Among the negative effects, it is mentioned the possible risks, such as zoonotic diseases proper of canine species^14^, difficulty to change residence or attend a stay for older adults, as pets are not allowed in these places and because they do not want to abandon them^18^. Other studies show that stress levels in people with canine companionship are higher as it involves expenses with veterinary and food, as well as care^21^. Finally, people with canine companionship do not show significant differences in terms of level of happiness, life satisfaction and physical performance when is compared to those who do not have a canine mascot^16^.

In short, it is observed that there is still a discrepancy between the effects produced by a canine companionship. It is clear that the human-animal bond can produce psychosocial well-being; however, it is necessary to continue conducting research to know the impact on the physical health in older adults. Therefore, the aim of this study was to compare the biomarkers and the allostatic load level in a sample of older adults with and without canine companionship.

## Method

This study was carried out using a quantitative, descriptive, comparative and cross-sectional research design. The study population consisted of older adults over 60 years of age, who lived in the community and attended a recreational center in the Comarca Lagunera (“region of lagoons”) in the states of Coahuila and Durango, Mexico.

The sample consisted of 106 adults over 60 years of age, distributed as follows: 53 with canine companionship and 53 without canine companionship. The sample was calculated using Epidat software version 4, according to the following parameters: 95% confidence interval and a power of 80%. A non-probability sampling or convenience sampling technique was used according to the following criteria: older adults with perception of time and space, without hepatic problems, and people who had heart attacks in the last 6 months and who had pets other than the canine species were excluded.

The sociodemographic data of the older adults, as well as their pets, were recorded on a background record and the data on the allostatic load were recorded on a medical chart.

Allostatic load measuring in older adults included 11 biomarkers, which are primary and secondary stress mediators. The measurement of cortisol was included as a primary mediator of the neuroendocrine system. Secondary mediators included: C-Reactive Protein (CRP) from the immune system; total cholesterol, High Density Lipoproteins (HDL) and glycosylated hemoglobin (Hb1Ac) from the metabolic system; systolic blood pressure (SBP) and diastolic (DBP) from the cardiovascular system and, finally, body mass index (BMI), waist circumference measurement, hip circumference measurement and waist-hip ratio (WHR) from the anthropometric system^7^.

The measurements of the biomarkers used to determine the allostatic load index were made as follows: an Omron arm digital baumanometer was used for arterial pressure. Blood pressure readings were taken three times, with a 2 minutes time difference between each reading, on the left arm, after the patient remain seated for 10 minutes and the readings were recorded in the medical chart. An average of the measurements was calculated to obtain a final systolic and diastolic blood pressure chart.

Weight measurement was performed using a digital scale of the brand Seca, appropriately calibrated. Similarly, height was measured in centimeters using a Seca stadiometer and the measurements were recorded in the medical chart. These data allowed the calculation of the Body Mass Index using Quetelet’s formula, in which the weight of the older adult, in kilograms, was divided by the square value of its height in meters (kg/m^2^).

A retractable fiberglass measuring tape was used for the waist and hip circumference measurements. Both measurements were recorded, in centimeters (cm), in the medical chart. These data were the basis for obtaining the Waist-Hip Ratio, which is calculate by dividing waist circumference by hip circumference.

A venous blood sample was analyzed to determine the CRP, total cholesterol, HDL, glycosylated hemoglobin and fibrinogen levels. This sample was collected and placed into its corresponding tube by trained personnel, stored in a cooler with coolants and transported to the laboratory for analysis, according to the following methods: turbidimetry for glycosylated hemoglobin, CRP, total cholesterol and HDL; fibrinogen was determined with a coagulometric method; and serum cortisol by chemiluminescence.

To calculate the allostatic load index, zero (low risk) was assigned to each biomarkers if they were within the normal cut-off points, and one (high risk) was assigned if they were above the reference values. Solely in the case of the biomarker HDL, zero (low risk) was assigned when its level was high, and one (high risk) when its concentration was low^24^, as shown in Table 1.

**Table 1:** Cut-off points for allostatic load biomarkers. Torreón, Coah, Mexico, 2017

Biomarker	Cutt-off point
Serum cortisol	> 25.0 μg/dl*
Total cholesterol	> 240 mg/dL^†^
High Density Lipoproteins	< 36 mg/dL^†^
Glycosylated Hemoglobin	> 7.1%^‡^
Fibrinogen	> 336 mg/dL^†^
C-Reactive Protein	> 0.3 mg/L^§^
Body Mass Index	> 25.0
Systolic blood pressure	> 148 mm/Hg^||^
Diastolic blood pressure	> 83.33 mm/Hg^||^
Waist Woman Man	> 85 cm^¶^ > 95 cm^¶^
Waist-hip Ratio	> 0.94

*μg/dl - Micrograms per deciliter; †mg/dL - Milligrams per deciliter; ‡% - Percentage; §mg/L - Milligrams per liter; ||mm/Hg - Millimeter of mercury; ¶cm - Centimeters

Finally, all biomarkers and anthropometric measurements were added with a possible range from 0 to 11. After performing the sum and because the population was composed of older adults, those with four or less altered biomarkers were classified as low risk and those with five or more altered biomarkers were classified as low risk^25^.

The relevant ethics and research committee approved this study under the protocol 2016/ELEUAC/001. After approval of the project, the relevant authorities have provided the permit for data collection. All participants signed an informed consent form. The older adults were informed as soon as the laboratory results were available, the results were communicate in writing and the recommendations for health care were provided.

For the statistical analysis, a database was created using SPSS v 20 software for Mac, descriptive statistics was applied as measures of central tendency and dispersion for quantitative variables, and relative frequencies for qualitative variables. For the comparison between groups, the Mann Whitney U test, Student’s t test and Chi-square were used, with a 95% confidence interval, and considering as significant *p<*0.05.

## Results

The older adults in the group with mascot had between 1 and 2 companion dogs (median of 1.5), with the small and medium dog breeds being the preferred ones in this sample, as 60% (32) had as companion the following dog breeds: Chihuahuas, French Poodle and Schnauzer mini, among others. The manner how the canines arrived at the older adults’ home in 70% (37) of the cases was as a gift from the family or a very close friend. They had an average of 5.5 years (SD=4.6) living with the older adult, and the dog lived most of the time outside the house, 37 (70%); however, the older adults mentioned that these pets had a free access to get into the house when the breeds were small, so that they could interact and play with them.

The sociodemographic characteristics of the older adults assigned to the groups with and without canine companionship are shown in Table 2. There was no significant difference between the characteristics listed, and there were groups with similar characteristics.

**Table 2:** Comparison between the sociodemographic characteristics of the groups with and without canine companionship. Torreón, Coah, Mexico, 2017

Sociodemographic characteristic	Older adult with canine companionship	Older adult without canine companionship	Statistical analysis
Age Mean Median SD^§^ Min-Max^||^	68.04 67.00 6.01 60-84	69.02 68.00 6.40 60-85	t*= -0.814, *df* ^†^=104, *p* ^‡^ *=*0.418
Education (Years of study) Media Median SD^§^ Min-Max^||^	6.89 6.00 4.41 0-18	7.38 6.00 5.03 0-25	t*= -0.534, *df* ^†^=104, *p* ^‡^ *=*0.594
Gender Woman Man	Frequency (%)^¶^ 42 (79.2) 11 (20.8)	Frequency (%)^¶^ 40 (75.5) 13 (24.5)	χ^2^**(1,n=106) = 0.215, *p* ^‡^= 0.408
Marital status With partner Without partner	Frequency (%)^¶^ 21 (39.6) 32 (60.4)	Frequency (%)^¶^ 27 (50.9) 26 (49.1)	χ^2^**(1,n=106) = 1.371, *p* ^‡^= 0.165
Presence of chronic disease(s) Yes No	Frequency (%)^¶^ 35 (66.0) 18 (34.0)	Frequency (%)^¶^ 43 (81.1) 10 (18.9)	χ^2^**(1,n=106) = 3.106, *p* ^‡^= 0.061

*t - Student’s t; †*df* - Degrees of freedom; ‡*p* - p-Value; §SD - Standard Deviation; ||Min-Max - Minimum Value - Maximum Value; ¶% - Percentage; **χ^2^ - Chi-squared

Amongst the 11 allostatic load biomarkers studied, cholesterol and cortisol showed significant difference in the groups with canine companionship and without canine companionship, with the highest mean found among those older adults who did not have a canine companion. The other allostatic load biomarkers showed a similar behavior in the study sample, without significant difference, as shown in Table 3.

**Table 3:** Comparison between the means of the allostatic load biomarkers in older adults with and without canine companionship. Torreón, Coah, México, 2017

Allostatic load biomarker	Older adult with canine companionship			Older adult without canine companionship			Statistics
	Mean	Median	SD*	Mean	Median	SD*	Student’s *t*
Total cholesterol	187.21	183.00	35.78	204.64	199.00	34.13	*t=* -2.566, *df* ^†^=104, *p* ^‡^ *=*0.012^§^
Serum cortisol	12.12	11.50	4.05	14.77	14.40	4.73	*t=* -3.091, *df* ^†^=104, *p* ^‡^ *=*0.003^§^
High Density Lipoproteins	57.15	58.00	13.10	55.38	54.00	12.68	*t=* 0.708, *df* ^†^=104, *p* ^‡^ *=*0.480
Glycosylated hemoglobin	6.40	5.90	1.82	6.83	6.10	1.79	*t=* -1.205, *df* ^†^=104, *p* ^‡^ *=*0.231
C-Reactive Protein	2.65	1.50	6.57	3.06	1.20	5.76	*t=* -0.338, *df* ^†^=104, *p* ^‡^ *=*0.736
Fibrinogen	468.25	450.00	97.11	438.36	441.00	80.54	*t=* 1.725, *df* ^†^=104, *p* ^‡^ *=*0.088
Body Mass Index	29.51	29.76	4.29	29.39	28.70	4.88	*t=* 0.125, *df* ^†^=104, *p* ^‡^ *=*0.901
Systolic Blood Pressure	140.91	135.67	27.32	147.33	140.00	29.69	*t=* -1.159, *df* ^†^=104, *p* ^‡^ *=*0.249
Diastolic Blood Pressure	76.84	73.33	15.58	78.99	78.00	13.31	*t=* -0.766, *df* ^†^=104, *p* ^‡^ *=*0.445
Waist-Hip Ratio	0.93	0.93	0.06	0.95	0.94	0.07	*t=* -1.149, *df* ^†^=104, *p* ^‡^ *=*0.253
Waist Women Men	97.83 101.55	97.50 106.00	9.79 10.82	100.75 98.96	102.00 101.00	9.19 14.43	*t=* -1.390, *df* ^†^=80, *p* ^‡^ *=*0.168 *t=* 0.488, *df* ^†^=22, *p* ^‡^ *=*0.630

*SD - Standard Deviation; †*df* - degrees of freedom; ‡p - p-value; §statistically significant value for p<0.05

**Figure 1:**
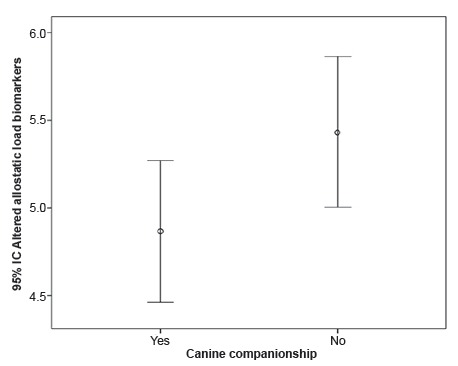
Mean and standard deviation of altered allostatic load biomarkers in older adults with (n=53) and without canine companionship (n=53): with companionship: mean= 4.87 + 1.47, without companionship: mean= 5.43 + 1.55. Mann-Whitney U test = 1714.00, Z= 2.01, *p=* 0.044

By comparing the allostatic load between the groups of older adults with and without canine companionship, a significant difference was observed between the groups studied as shown in Figure 1.

Finally, when comparing allostatic load levels and canine companionship, a higher frequency of older adults with low allostatic load was found among those who had companion dog, with a significant difference, as shown in Table 4.

**Table 4:** Comparison between the allostatic load levels in older adults and canine companionship. Torreón, Coah, Mexico, 2017

	Low Allostatic Load		High Allostatic Load	Total	Statistics
Canine companionship	Yes	20 (38%)	33 (62%)	53 (100%)	χ^2^ = 3.69*, *df*=1^†^, *p*= 0.043^‡^
	No	11 (21%)	42 (79%)	53 (100%)	
Total		31 (29%)	78 (71%)	106 (100%)	

*χ^2^ - Chi squared; †*df* -degrees of freedom; ‡p - p-value

## Discussion

In this study, it was observed that older adults tend to have small species as canine companion, since it is easier for an older person to transport his dog to the veterinarian or provide care for him at home, such as bathing, feeding and walking. In addition, small species poses a lower risk for falls in the older adult at home.

The canine companion comes to the older adult’s life mostly as a gift, which is closely linked to the social and emotional aspect of those who value an older person. In other words, they decide to provide a canine companion to an older adult to avoid him to feel lonely, also to make him to dedicate part of his time to activities of care and recreation through having a mascot^4^.

Other studies report that 67% of mascots sleep and spend more time outside the house^15^, similarly to this study, in which 70% of mascots stay outside the house of the older adult. However, the difference is that the older adults mentioned that the pet has a free access to get into the house as part of the interaction and care for the canines.

Cholesterol, as a secondary biomarker of the metabolic system, had a significant difference in older adults who have a canine companionship, compared to those who did not have. As reported in another Latin American study^21^, this significant difference may be influenced by the time that older adults spend walking their pets^17^ ^-^ ^19^, as well as by the need of feeling good and being able to care for their pets^20^.

Similar to the results found in other researches, in this study, the cortisol levels in older adults who have a canine companionship are lower than in those who do not have a canine companionship^19^ ^,^ ^21^ ^,^ ^23^. The explanation for this difference lies on the findings of an international author who observed that there is a lower perception of stress in those adults over 60 years who have a canine companionship^11^, and cortisol, as a primary mediator, is a hormone that is released when a person is exposed to stressful situations.

Unlike other studies showing that older adults with canine companionship have a lower systolic blood pressure^11^ ^,^ ^21^, the result of this study shows no significant difference in this biomarker, so there is no evidence to determine the effect on this variable.

Finally, there are no previous studies on the association between canine companionship and allostatic load level, and it is demonstrated in this research that older adults without canine companionship have higher allostatic load levels than those who have the companion of this species. The high allostatic load level was estimated based on altered biomarkers and, therefore, it can be stated that older adults without canine companionship have more than 5 biomarkers classified as high risk, presenting an increased possibility to have health complications.

The scope of this study is limited as a descriptive and comparative design was used, and it is recommended for future studies the use of approaches for a more effective control of the external variables that may affect the results. In addition, an in-depth analysis of the variable “attachment to pet” is proposed, as this variable could modify the results, and it was not included in the present study.

## Conclusion

The canine companionship has an association with cortisol and cholesterol levels in older adults, as the levels of these biomarkers are lower when compared to those found in older adults without canine companionship. These biomarkers play an important role in the control and maintenance of health, as well as in the development of allostatic load in older adults.

Sometimes, the mascot arrives unexpectedly at home or as a gift for a loved one, but this can have a positive impact on the physical health of the older adult, resulting in a new field of action in nursing that aims to encourage and create new strategies to promote health. Finally, the canine companionship could have a mediating effect on the psychosocial stress in older adults.
